# Upper Trunk Brachial Plexus Palsy Following Chiropractic Manipulation

**DOI:** 10.3389/fneur.2016.00211

**Published:** 2016-11-30

**Authors:** John Cunningham, Wayne Hoskins, Scott Ferris

**Affiliations:** ^1^Department of Orthopaedic Surgery, Royal Melbourne Hospital, Parkville, VIC, Australia; ^2^Faculty of Medicine, Dentistry and Health Sciences, The University of Melbourne, Parkville, VIC, Australia; ^3^Victorian Plastic Surgery Unit, St Vincent’s Private Hospital, East Melbourne, VIC, Australia

**Keywords:** brachial plexus injury, upper trunk, Erb’s palsy, manipulation, chiropractic

## Abstract

**Introduction:**

Upper trunk brachial plexus palsy can result from high-energy trauma and has never been reported following spinal manipulation.

**Background:**

The case is presented of a patient who developed an acute brachial plexus upper trunk palsy following spinal manipulative therapy.

**Discussion:**

Discussion is made on the incidence of complications following manipulation and recommendations to prospectively capture all serious complications.

**Concluding remarks:**

Risks exist with spinal manipulative therapy. Neurological injury can occur. Risk assessment and re-examination should occur at every visit. Large rigorous prospective studies are required to identify the true incidence of serious complications resulting from manipulative therapy and the benefit:risk ratio.

## Introduction

Traction injury to the upper trunk of the brachial plexus, or its constituent C5 and C6 nerve roots, is a common cause of altered function of the plexus. In children, it most commonly follows birth trauma due to shoulder dystocia (1). In adults, the mechanisms include high speed motor bike accidents, high speed car accidents, water-skiing accidents, as well as claviclular fractures, traumatic falls, or contact collision sporting injuries (2, 3). The palsy classically results in the patient adopting the “waiter’s tip” position with the arm adducted and the shoulder internally rotated, the elbow extended and with the forearm pronated and the fingers and wrist flexed. Sensory deficits occur over the lateral shoulder and preaxial upper limb, and the injury can be partial or complete. The prognosis is variable, and timely expert assessment and appropriate interventions are critical to maximize long-term limb function. Isolated C5,6 brachial plexus palsy, referred early is, with contemporary techniques, an entirely treatable problem. To the best of our knowledge, no case of iatrogenic upper trunk brachial plexus palsy has been previously documented following spinal manipulative therapy.

## Background

The patient was a 36-year-old man educated to year 10. He provided written consent for presentation of this case report. Since 2010, he had worked on a commercial shark fishing boat and prior to this worked on a dairy farm for 3 years. His typical work duties on the boat involved pulling sharks out of a large net and gutting them on a table and trips away would typically last between 10 and 14 days.

In 2000, he suffered a lower back injury and had a lumbar fusion that same year. This was revised, and rods were taken out in 2004. He had been working without any problems with his lower back since that time.

The patient started to see a chiropractor because of the persistent tightness and soreness that he experienced in his hands, arms, and shoulders present since he was 15. It was made worse with physical labor, and he found some relief with tiger balm, hot showers, and paracetamol. He had not had any previous sporting trauma to his neck. Chiropractic treatment involved the “Activator” device (a handheld spring loaded instrument that delivers a high-velocity low-amplitude force) and various stretching and soft tissue massage techniques that resulted in temporary relief as well.

In 2003, he started to develop pins and needles in his right hand, and to a lesser extent his left hand. The pins and needles were situated in his thumb and index finger. Symptoms were aggravated on fishing trips, and he would often see his chiropractor after these trips.

His second last fishing trip finished on December 9, 2010, and he went to see his chiropractor 4 days later. The patient told the chiropractor that his right hand pain was deteriorating. The chiropractor, after using the “Activator,” then proceeded to a manual spinal manipulative therapy technique.

The patient describes lying prone with his head turned to the left, with the chiropractor’s arms underneath the patient’s armpits, and the closed hands behind the patient’s head. A maneuver that forcibly increased the angle between his head and his shoulders was performed. The patient felt pressure in the middle of his back which he thought was the chiropractor’s knee. He then felt a jolt with sudden onset of a burning sensation in his left arm. This burning sensation lasted for about 30 s, and the patient reported it to the chiropractor.

The patient noticed weakness when he went to pay for his appointment. He was unable to actively lift his left hand up to the counter top. He had to use his right arm to grasp his left wrist to elevate it. His left hand was still able to hold and manipulate his wallet.

The patient reported that over the next couple of days the weakness did not change, and he returned to the chiropractor 2 days later. He was reassured by the chiropractor and was instructed that he should be fine to go out onto the fishing trip that was leaving that day.

The patient went out to sea but unfortunately was unable to perform his usual duties. It became evident that he was unable to elevate his left arm or control it with any dexterity. Upon return, he immediately went to see his general practitioner who managed the patient and eventually referred for surgical opinion and management in 2014.

The patient’s complaints when seen by the authors in July 2014 were still predominantly of weakness in his left arm associated with cramping and numbness. He states that his left arm “does not work.” He is unable to actively lift his left hand from its hung position. He experiences pain which starts in the middle of his neck and radiates into his left trapezius and down to between his shoulder blades. He had tried a shoulder brace in the past without significant benefit.

Functionally, he has difficulty in dressing himself, combing his hair, and hugging his partner. He found it difficult to get dressed by himself, and when he eats, he has to lift his left arm and place it on the table. He has not noticed any problems with dexterity or power within his actual hand, but he is unable to move his hand away from the side of his body. He has been unable to work on the fishing boat since.

Previously, the patient was well and on no medications. He smoked a packet of cigarettes a day and was taking Jurnista, Lyrica, and Mobic. He has no known allergies.

**Figure 1:**
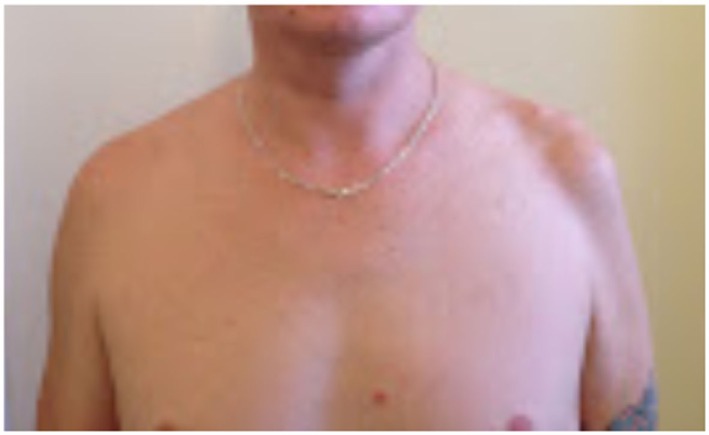
**Antero-posterior view**.

**Figure 2:**
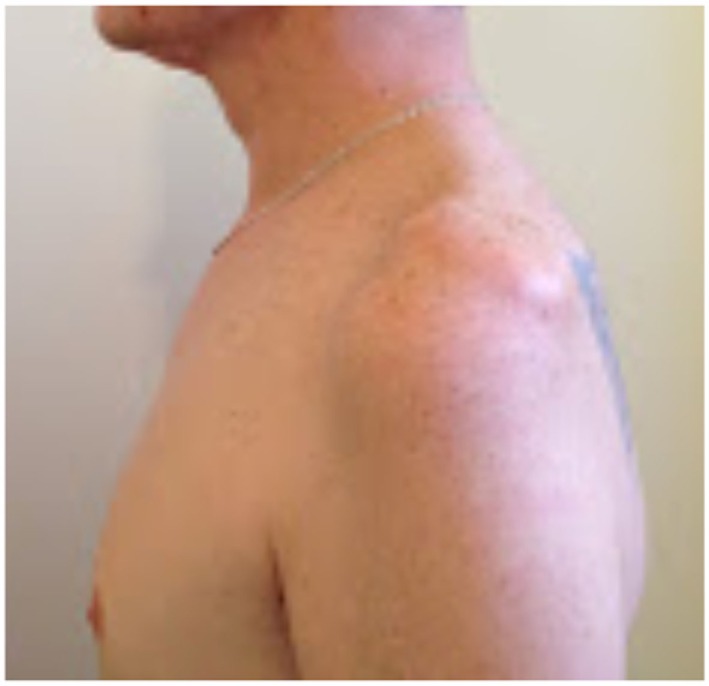
**Left lateral view**.

**Figure 3:**
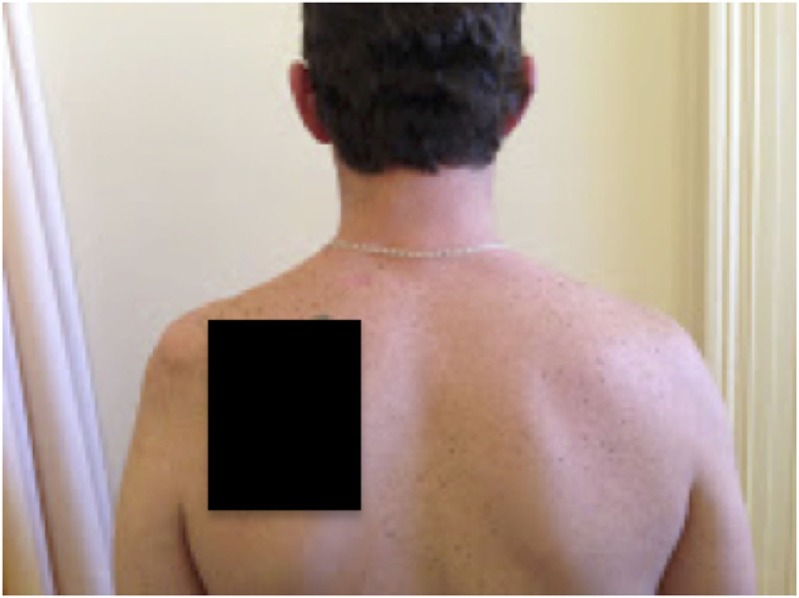
**Postero-anterior view 1**.

**Figure 4:**
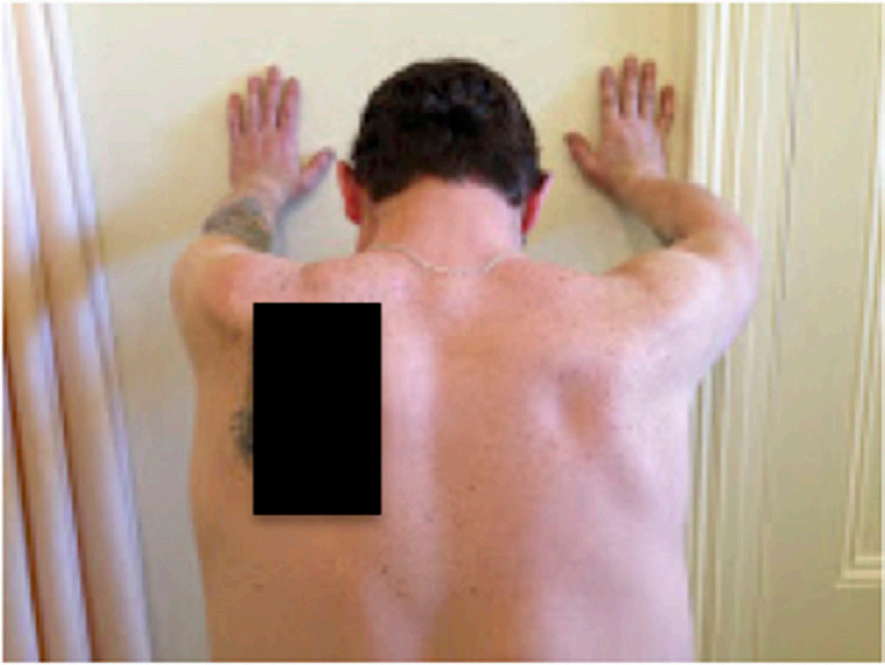
**Postero-anterior view 2**.

**Figure 5:**
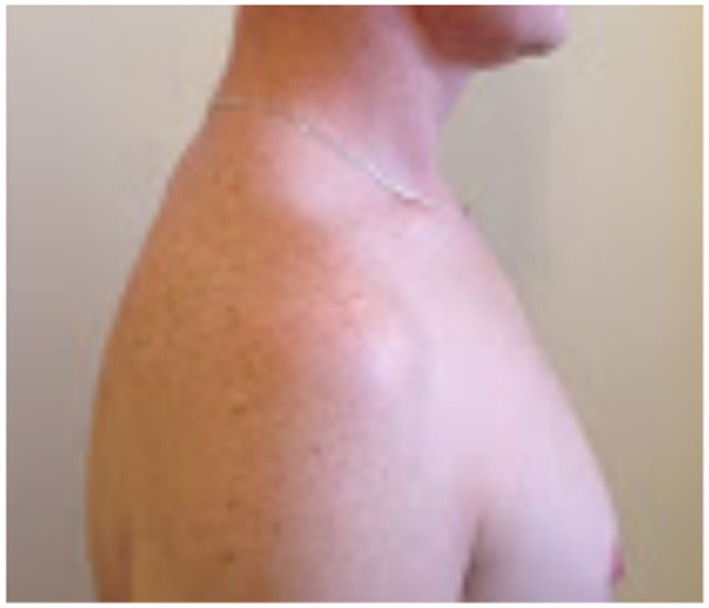
**Right lateral view**.

On examination, the patient’s left arm remained by his side in a pronated position. He had gross wasting of his left shoulder girdle and his left biceps (see Figures 1–5). Throughout the examination process, he was unable to move his left shoulder or flex his elbow with any observed strength. His left arm had decreased biceps jerk and brachioradialis reflex, while his triceps jerk was intact. His shoulder, elbow, and wrist had a full passive range of motion. He had normal power of internal rotation of his left shoulder but weakened external rotation. He had Grade 0 power of his biceps, brachioradialis, and deltoids. He had Grade V power of pronation but Grade II power of supination. His triceps power was Grade V and all groups including and distal to the wrist were Grade V. He had decreased sensation to light touch around his shoulder region and down to his thumb. His cervical spine exhibited a normal range of motion. He had good distal pulses. His right arm was neurologically intact.

He had a well-healed surgical scar in his lumbar spine. He was able to walk with a normal gait.

**Figure 6:**
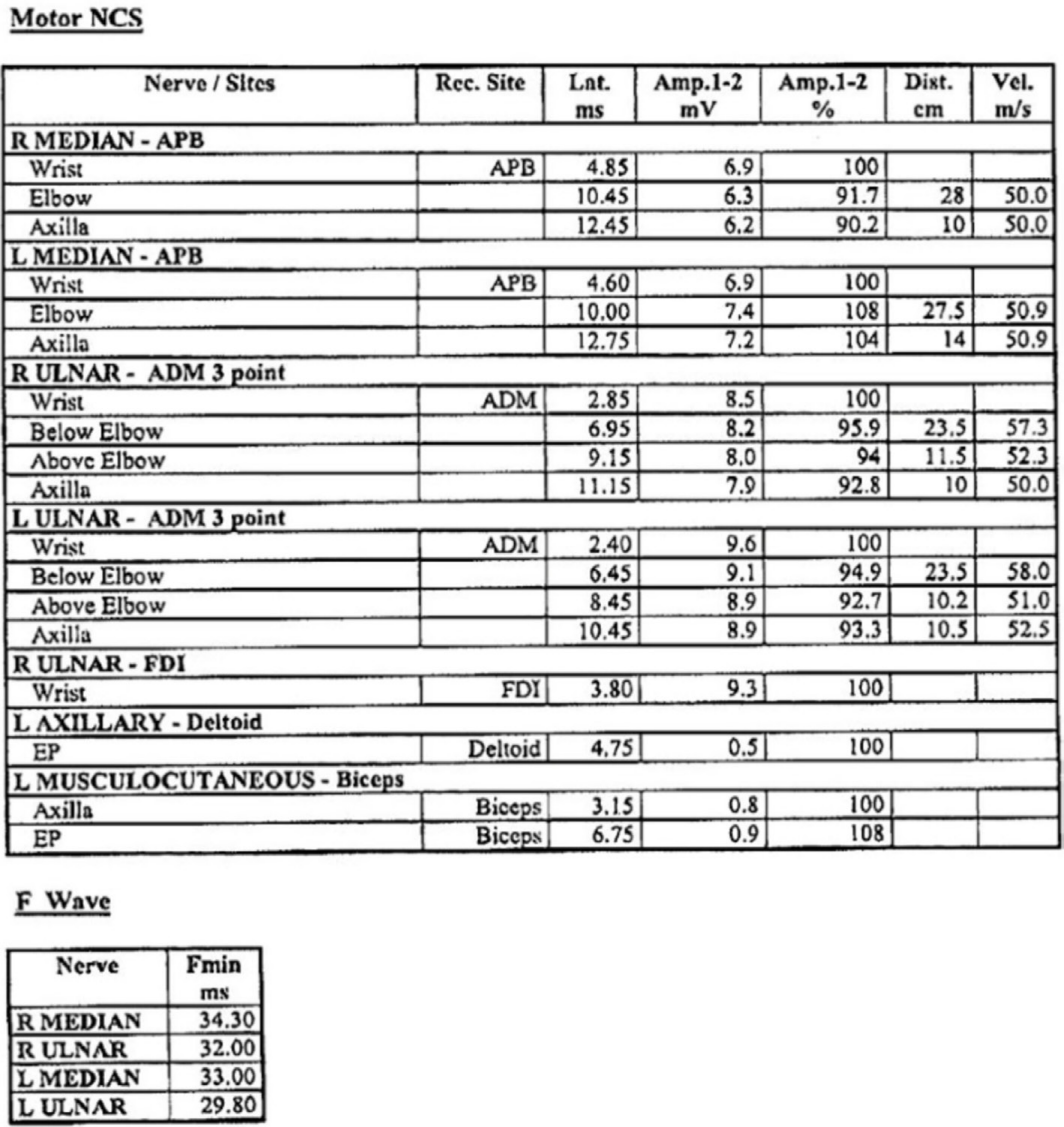
**Motor Nerve Conduction Studies**.

**Figure 7:**
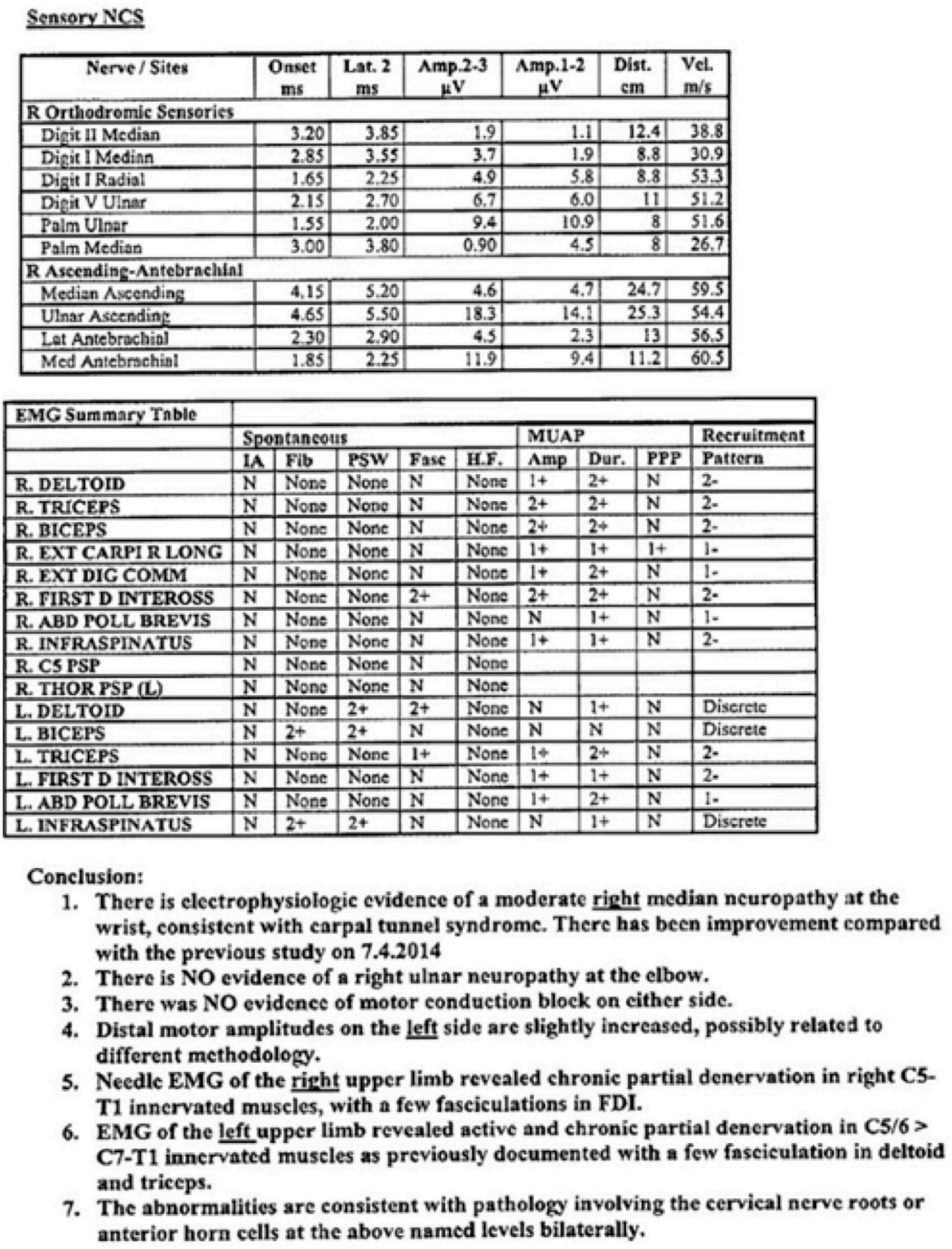
**Sensory Nerve Conduction Studies**.

An MRI had been performed in April 2014, which revealed mild degeneration throughout most of his cervical spine and loss of disc height at C6/7. Bony foraminal stenosis was evident at C4/5 on the left and C5/6 bilaterally. There was no evidence of root avulsion. Nerve conduction studies revealed active and chronic partial denervation in the C5/6 innervated muscles with a few fasciculations in the deltoid and triceps in the left arm, and evidence of carpal tunnel syndrome on the right (Figures 6 and 7). These abnormalities were consistent with pathology involving the cervical nerve roots or anterior horn cells.

Our impression was that the patient was suffering from a left traction injury to his cervical spine, upper trunk brachial plexuses injury, or possibly a double crush type injury. His symptoms and physical findings were most consistent with an Erb-Duchenne palsy.

Currently, the patient is undergoing a series of reconstructions, utilizing free functioning muscle transfer and regional tendon transfers, in stages, to restore elbow flexion, external rotation, and abduction. The complex and staged surgery and rehabilitation will, by necessity, take 2–3 years but is very likely to result in significant restoration of function. Earlier referral would have been desirable with reconstruction commencing well within the first year after injury. The rehabilitation phase required when nerve transfer reconstruction is possible can be as short as 1 year. Overall then, this patient would have had functional restoration approximately 4 or 5 years earlier had timely referral and surgery occurred. Additionally, the best result from early nerve transfer surgery is significantly superior to the best result from late salvage free and regional muscle transfers.

## Discussion

It is clear that the original complaint in the right upper limb was carpal tunnel syndrome, a relatively easily diagnosed and treatable condition. The case report, and specifically the temporal relationship of the maneuver to the acute palsy symptoms, suggests the lesion to be either a pre-existing condition exacerbated by manipulation or trauma resulting from the manipulation itself. The hypothesized mechanism of injury in this case involved a high-velocity distraction force with widening of the angle between the head and shoulder. The authors have not been able to define the maneuver according to conventional chiropractic techniques. This mechanism would appear to be a similar mechanism to high-energy sporting injuries such as that which occur in football (4) or motor vehicle accidents (5), producing injury to nerve roots or brachial plexus and a spectrum of injury from reversible neuroraxia to permanent injury. In these situations, the upper trunk is the most commonly injured (5). Cervical spine radiculopathies have been reported to develop from repeated sporting cervical spine trauma (6) and may be more common with congenital or acquired narrowing of the spinal cord (7).

Chiropractic and manual therapy literature states that manipulative therapy is contraindicated in the acute phase of a disc or nerve root injury (8). A thorough history and physical examination should occur on injured patients including neurological and provocation-based testing which will determine contraindications for manipulative therapy and referral for advanced imaging and other specialist services if required (9, 10). Recognition of carpal tunnel syndrome by his treating chiropractor and appropriate referral may have provided the patient a more rapid return to work and have avoided this complication.

Risk assessment and re-evaluation for rational continuation of treatment should be performed at every visit (11). Doing so can prevent an estimated 44.8% of adverse events associated with cervical spine manipulative therapy (12). It is recognized though that some chiropractors choose not to do this (13). All chiropractors and manual therapists should be familiar with risk management for patient safety and should implement it actively in the provision of care (14). This may result in appropriate patient screening and selection for treatment choice and modality. It has been estimated that 10.4% of adverse events from cervical spine manipulative therapy are unpreventable (12).

Chiropractors have a range of different treatment modalities besides spinal manipulative therapy (10). Chiropractic practitioners who exercise a unimodal, manipulation-only approach do so despite undergraduate university education and training. Guidelines for the selection of a chiropractor have been proposed (10). Successful reports have been published with a change in treatment paradigm to not include spinal manipualtion with the development of neurological symptoms, including surgical referral (6).

Complications resulting from spinal manipulative therapy are known to occur (15–18) and are not limited to chiropractic management (19). Most complications are transient and self-resolving. More serious neurological complications have been known to occur such as radiculopathy and myelopathy (9, 20). However, cause and effect cannot be definitely attributed in all cases due to the natural history of herniated disc and stenosis (21). A 6-year retrospective review of patients presenting to a neurosurgical practice who developed neurological deterioration after spinal manipulative therapy identified 18 cases (9). Another 5-year retrospective review found 22 patients presenting for deteriorated following cervical manipulative therapy (20). Discrepancies may exist between what was reported and what actually occurred as the authors dealing with the effects of the adverse event publish the cases rather than the therapist (12).

The time from injury to definitive surgical review in this case was approximately 4 years, and as such the opportunity for direct neurotisations or reinnervation of the patient’s native biceps, brachialis, supraspinatus, infraspinatus, and deltoid muscles had been lost. In this delayed situation, the most reliable techniques for restoration of lost motor function depend on tendon transfers, free functioning muscle transfers, and/or shoulder arthrodesis (22).

## Concluding Remarks

Determining an ideal study design to capture the true incidence of more serious events resulting from spinal manipulation is challenging. A prospective national survey that obtained data from 50,276 cervical spine manipulations reported no serious adverse events (23). Large rigorous prospective registries of adverse events, such as exist in medicine and surgery, would seem the best design, but cost, implementation, and achieving a high response rate remain the challenge (24). Until this occurs, the true incidence of serious complications resulting from manipulative therapy and the benefit:risk ratio remains unknown.

## Author Contributions

JC and SF contributed the clinical case. All the authors participated in drafting and revising the work, approved the final version to be published, and agreed to be accountable for all aspects of the work.

## Conflict of Interest Statement

The authors declare that the research was conducted in the absence of any commercial or financial relationships that could be construed as a potential conflict of interest.
